# Supplementary material for: Wnt5A and TGFβ1 Converges through YAP1 Activity and Integrin Alpha v Up-Regulation Promoting Epithelial to Mesenchymal Transition in Ovarian Cancer Cells and Mesothelial Cell Activation
Source: Cells. 2022 Jan 11;11(2):237. doi: 10.3390/cells11020237 (PMC8773996; doi:10.3390/cells11020237)
Supplement: Supplementary file 1 [file cells-11-00237-s001.zip › cells-1439091-supplementary.pdf]

Table S1 : Primer sequences used for qRT-PCR

Gene name	Sequence
ACTB	F: 5´-CTTCCTTCCTGGGCATG-3'<br>R: 5´-GTCTTTGCGGATGTCCAC-3'
CDH-1	F: 5´-GCTCTCCACTCTTACTTCCT-3´<br>R: 5´-GTTTGGTCTGATGCG-3´
CDH-2	F: 5´-GCCCAAGACAAAGAGACCC-3´<br>R: 5´-CTGCTGACTCCTTCACTGAC-3´
WNT5A	F: 5'- GCCATGAAGAAGTCCATTG -3'<br>R: 5'- AGCGACCACCAAGAATTG-3'
ROR-2	F: 5'- AATCACAGCGGCCTTCACC-3'<br>R: 5'- GGCACAGGTCGCTCTCCA-3'
ROR-1	F: 5'- TAATCGGAGAGCAACTTCA-3'<br>R: 5'- TGTAGTAATCAGCGGAGTAA-3'
TGFB1	F: 5'- AACCCACAACGAAATCTATGAC-3'<br>R: 5'- TAACTTGAGCCTCAGCAGAC-3'
TGFB2	F: 5'- CCATCCCGCCCACTTTCTAC-3'<br>R: 5'- AGCTCAATCCGTTGTTCAGGC-3'
TGFBR1	F: 5'- CACAGAGTGGGAACAAAAAGGT-3'<br>R: 5'- CCAATGGAACATCGTCGAGCA-3'
TGFBR2	F: 5'- AAGATGACCGCTCTGACATCA-3'<br>R: 5'- CTTATAGACCTCAGCAAAGCGAC-3'
YAP	F: 5'- TGTCCCAGATGAACGTCACAGC-3'<br>R: 5'- TGGTGGCTGTTTCACTGGAGCA-3'
TAZ	F: 5'- ACCGTGTCCAATCACCAGTCCT-3'<br>R: 5'- CCTTGGTGAAGCAGATGTCTGC-3'
FAP	F: 5'-GGAAGTGCCTGTTCCAGCAATG-3'<br>R: 5'-TGTCTGCCAGTCTTCCCTGAAG-3'
CCN1	F: 5'- CGAGGTGGAGTTGACGAGAA-3'<br>R: 5'-GAGGCTCCATTCCAAAAACAGG-3'
CCN2	F: 5'- CTTGCGAAGCTGACCTGGAAGA-3'<br>R: 5'- CCGTCGGTACATACTCCACAGA-3'
ACTA2	F: 5'- CTATGCCTCTGGACGCACAACT-3'<br>R: 5'- CAGATCCAGACGCATGATGGCA-3'
SNAI1	F: 5'- CCAGAGTTTACCTTCCAGCA-3'<br>R: 5'- GATGAGCATTGGCAGCGA-3'
SNAI2	F: 5'- AACTACAGCGAACTGGACAC-3'<br>R: 5'- GGATCTCTGGTTGTGGTATGAC-3'
FN1	F: 5'- ACCACATCGAGCGGATCTG-3'<br>R: 5'- TCTGTGACACAGTGGCCATAGG-3'
VIM	F: 5'- GGCTCGTCACCTTCGTGAAT-3'<br>R: 5'- GAGAAATCCTGCTCTCCTCGC-3'
CD44	F: 5'- CTGCCGCTTTGCAGGTGTA-3'<br>R: 5'- CATTGTGGGCAGGGTGCTATT-3'
ITGB8	F: 5'- CTGTTTGCAGTGGTCGAGGAGT-3'<br>R: 5'- TGCCTGCTTCACACTCTCCATG-3'
ITGB6	F: 5'- TCTCCTGCGTGAGACACAAAGG-3'<br>R: 5'- GAGCACTCCATCTTCAGAGACG-3'
MSLN	F: 5'- CCTGAGGACATTCGCAAGTGGA-3'<br>R: 5'- CTTCCCTTCACAAAGCGGTCGA-3'
KRT 18	F: 5'- GGAGGCATCCAGAACGAGAA-3'<br>R: 5'- CCAGCTGCAGTCGTGTGATA-3'
ITGAV	F: 5'- GCAACAGGCAATAGAGAT-3'<br>R: 5'- TGCTGAATCCTCCTTGACAA-3'
CALB2	F: 5'- AGCGCCGAGTTTATGGAGG-3'<br>R: 5'- TGGTTTGGGTGTATTCCTGGA-3'

Table S2: Clinicopathological characteristics of patients

Tumor grade	Number of sample	Median age	Treatment	Time of sample collection (after diagnosis) Patient
Normal ovary	6	42	_	_
BLSOC	10	48	None	AD
LGSOC (Grade I,II)	10	53	None	AD
HGSOC (Grade III,IV)	15	57	None	AD

AD: After diagnosis, before treatment. Borderline serous ovarian cancer (BLSOC, low-grade serous ovarian cancer (LGSOC), high-grade serous ovarian cancer (HGSOC).

Table S3. Common GO (BP) and Protein-protein interaction network for Wnt5A and TGF-b signaling components

GO.ID	Term	P value
GO:0001837	epithelial to mesenchymal transition	0.0019
GO:0071560	cellular response to transforming growth factor beta stimulus	0.00505
GO:0048762	mesenchymal cell differentiation	0.0021
GO:0071363	cellular response to growth factor stimulus	0.007
GO:0043406	positive regulation of MAP kinase activity	0.0089
GO:0032147	activation of protein kinase activity	0.0117
GO:0042060	wound healing	0.00255
GO:0045596	negative regulation of cell differentiation	0.00755
GO:0008284	positive regulation of cell proliferation	0.02125
GO:0001501	skeletal system development	0.00735
GO:0010468	regulation of gene expression	0.0519
GO:0010628	positive regulation of gene expression	0.0386
GO:1902533	positive regulation of intracellular signal transduction	0.024
GO:1903508	positive regulation of nucleic acid-templated transcription	0.02515
GO:0045893	positive regulation of transcription, DNA-templated	0.05605
GO:0006355	regulation of transcription, DNA-templated	0.07995
GO:0010604	positive regulation of macromolecule metabolic process	0.01385
GO:0051240	positive regulation of multicellular organismal process	0.1015
GO:0030182	neuron differentiation	0.007
GO:0043009	chordate embryonic development	0.00285

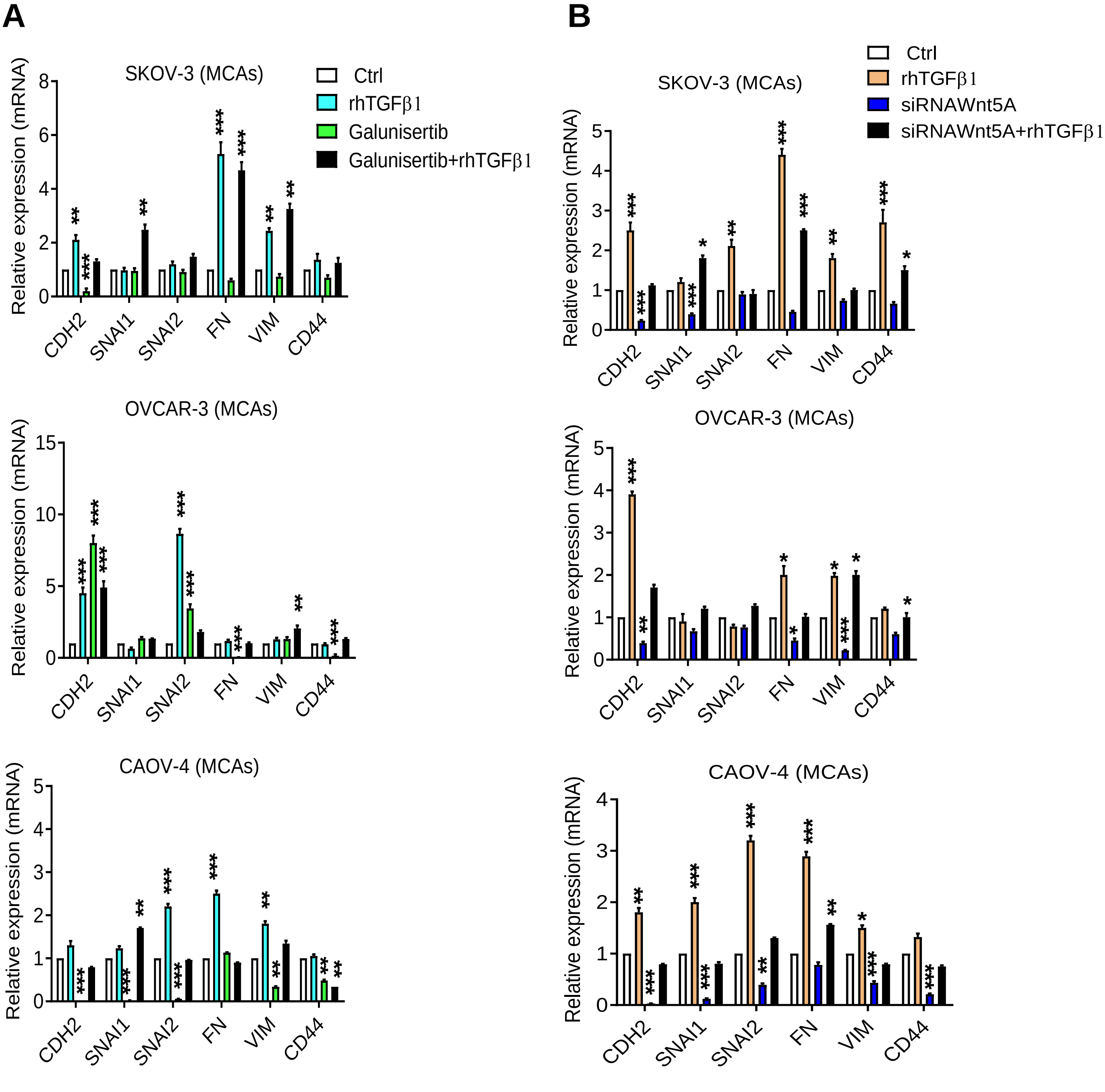

Figure S1. The expression levels of EMT markers in the presence of rhTGFβ1, Galunisertib and Wnt5A knocked-down OvCa cells alone or in combination. (A) Cells were treated with Galunisertib (10 μM) for 48h and rhTGFβ1 (10ng) for 1h alone or in combination. **(B)** Untreated and siRNA Wnt5A or scrambled transfected cells in the absence or presence of rhTGFβ1 (10ng) for 1h. Data were normalized related to ACTB expression levels as an internal control and presented as mean ± SD, n=3. *: $P < 0.05$, **: $P < 0.01$ and ***: $P < 0.001$ compared to scramble (Scr), n=3. MCAs: multicellular aggregates

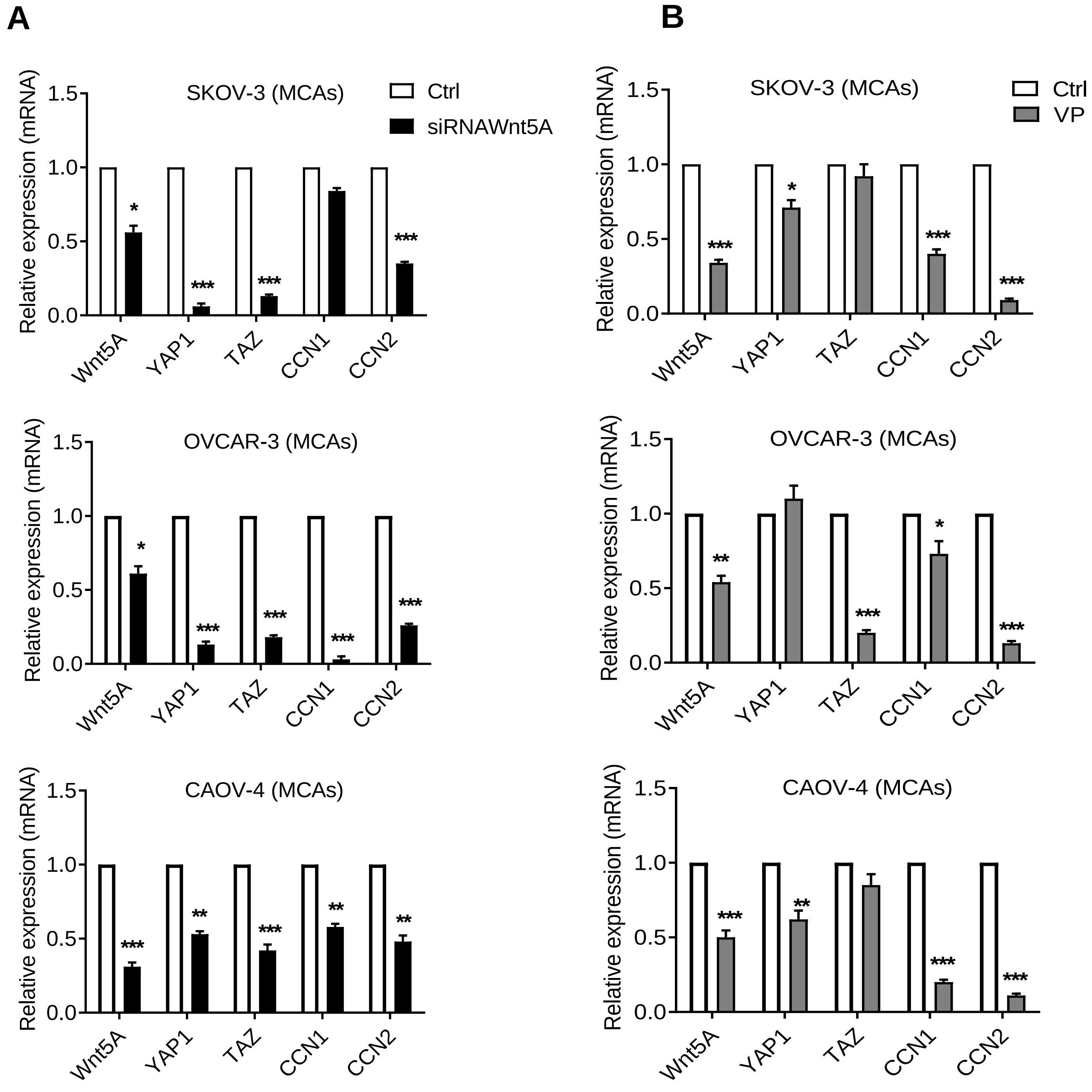

Figure S2. Wnt5A regulates YAP1 and its downstream targets. The mRNA levels of Wnt5A, YAP1, TAZ, CCN1 were assessed by the qRT-PCR assay in **(A)** siRNA Wnt5A multicellular aggregates (MCAs) cells or Scr transfected treated. **(B)** Verteporfin (VP)-treated MCAs cells (5 μM for 1h for SKOV-3 and OVCAR-3 and 2h for CAOV-4 cells). Data were normalized related to ACTB expression levels as an internal control. *: *P* < 0.05, **: *P* < 0.01 and ***: *P* < 0.001 compared to scramble (Scr), n=3. MCAs: multicellular aggregates

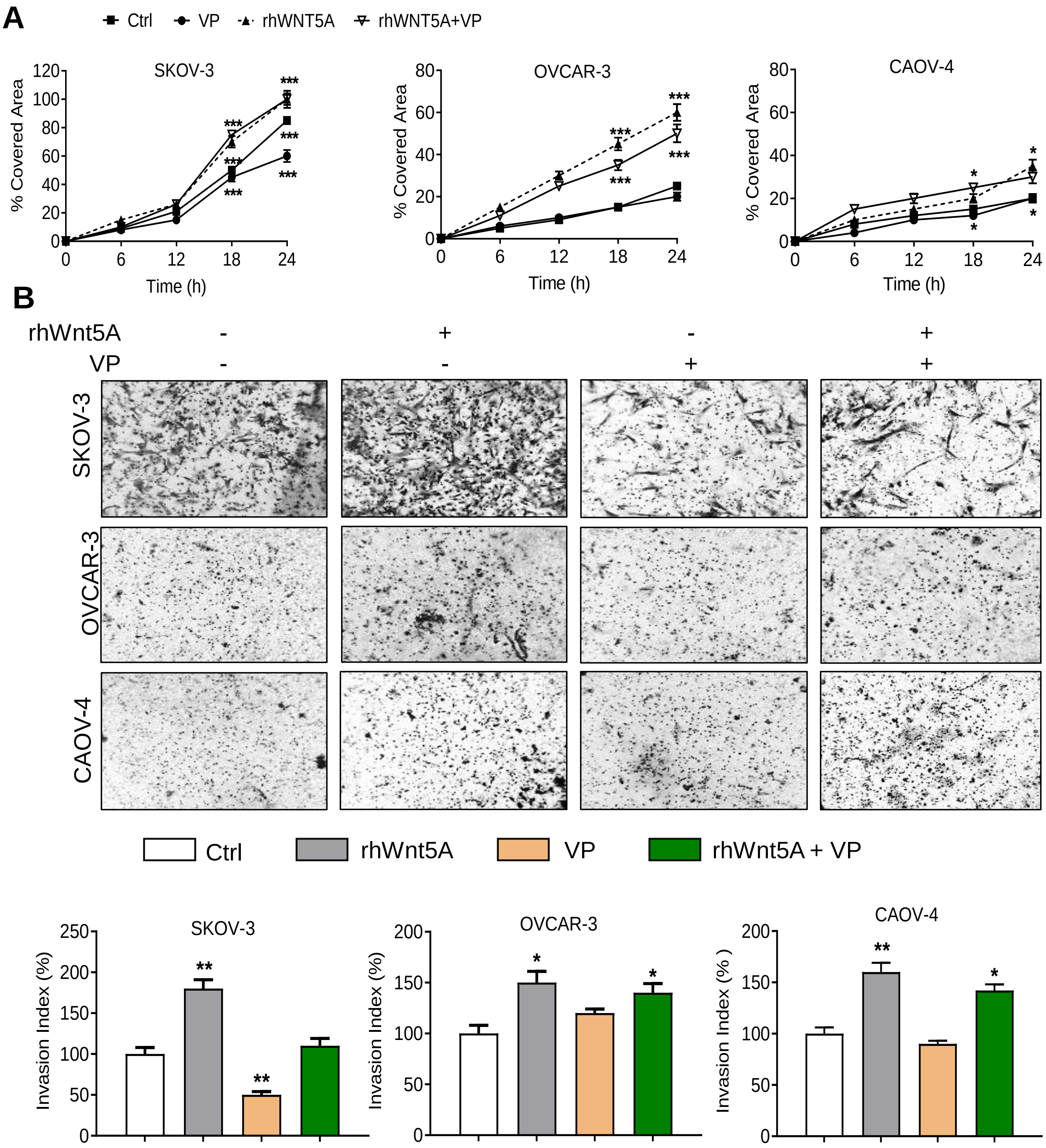

Figure S3. Verteporfin alters cell migration and invasion of OvCa cells. The cells were pre-treated with Verteporfin (VP) (5 μM) or treated with rhWnt5A (600 ng/ml) alone, or VP pretreated + rhWnt5A and **(A)** Wound healing was performed, and the percent of the covered area was quantified for the indicated times. (**B)** Invasion assay was performed using Matrigel-coated transwells. Upper panel: Photos represent one of the three independent experiments. Original magnification: ×100. Lower panel: The number of invaded cells was quantified as described in materials and methods. Results were presented as mean ± SD, n=3. *: $P < 0.05$, **: $P < 0.01$ and ***: $P < 0.001$ compared to untreated control cells (Ctrl).

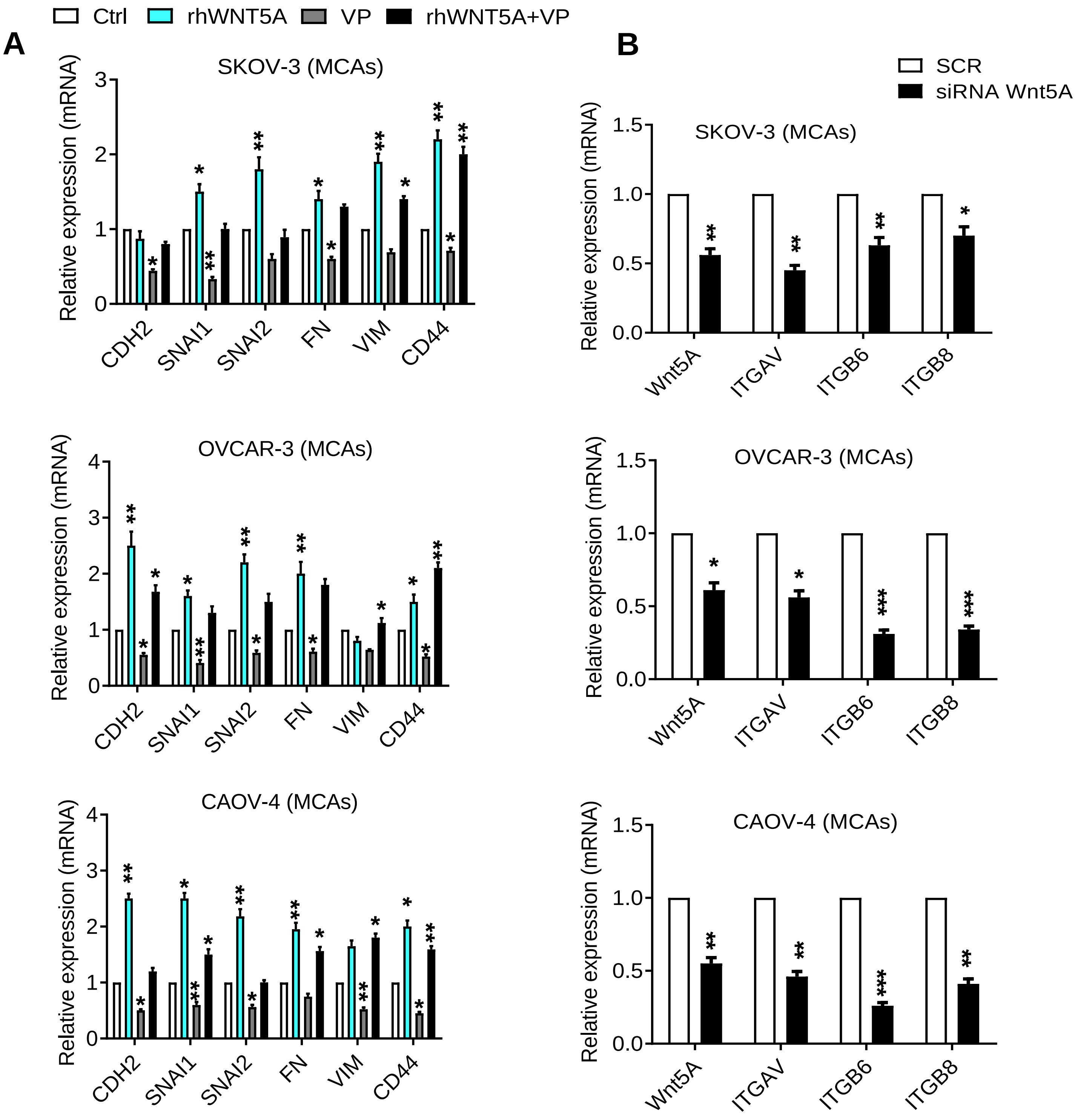

Figure S4. Regulation of the expression levels of EMT markers and integrins in OvCa cells. **(A)** EMT-related genes were assessed by qRT-PCR analysis in the presence of verteporfin (VP) (5 μM) or rhWnt5A (600 ng/ml) alone or in combination. **(B) C**ells were transfected with siRNA Scrambled (Scr) or siRNA against Wnt5A. The mRNA expression levels of integrin- alpha v (ITGAV), -beta6 (ITGB6), and -beta8 (ITGB8) were assessed by qRT-PCR analysis. Data were normalized related to ACTB expression levels as an internal control. *: $P < 0.05$, **: $P < 0.01$ and ***: $P < 0.001$ compared to scramble (Scr), n=3. MCAs: multicellular aggregates

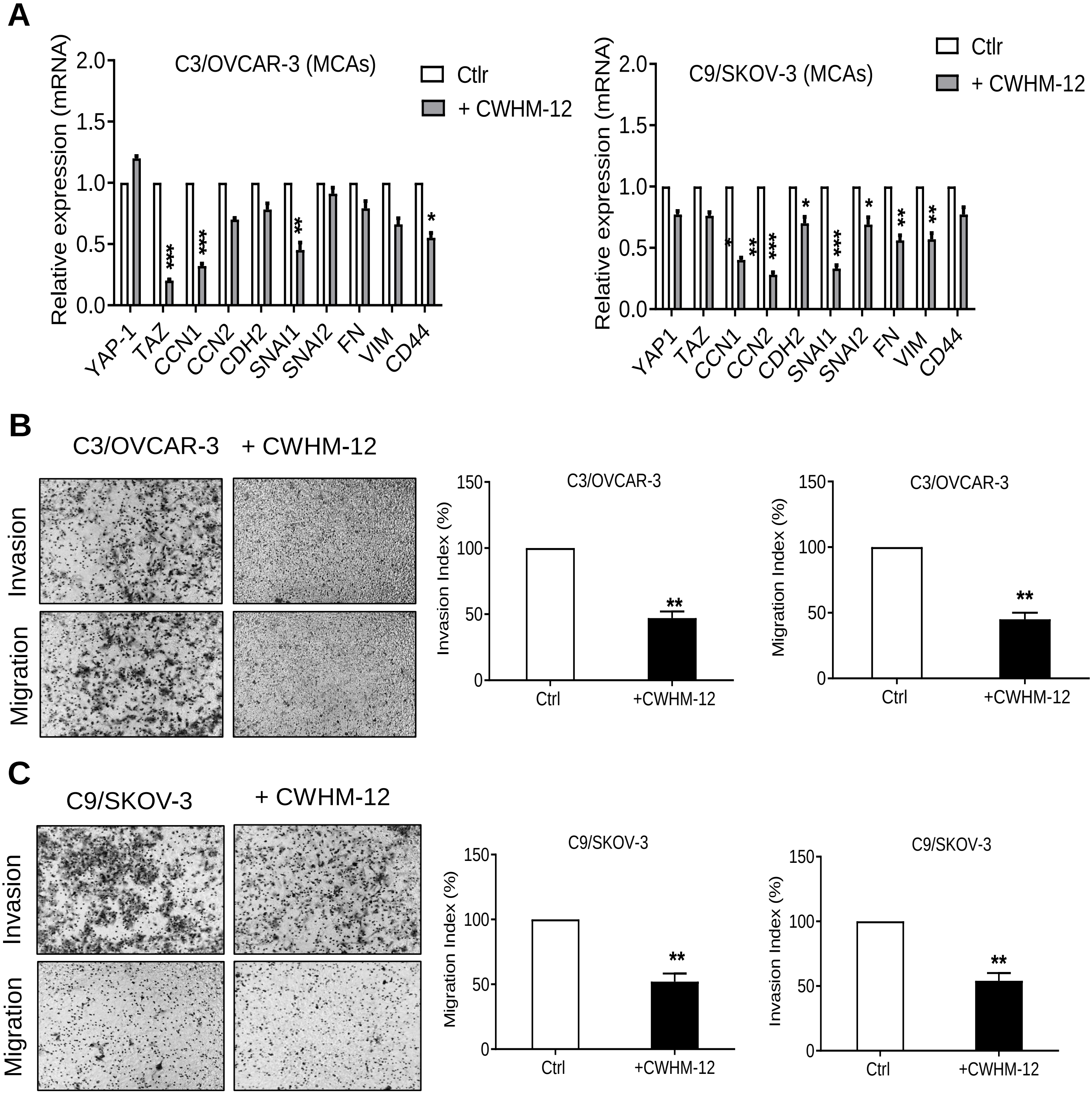

Figure S5. Expression of EMT markers, migration, and invasion of Wnt5A overexpressed OvCa cells in the presence of CWHM-12. The OVCAR-3 clone (C3/OVCAR-3) and SKOV-3 clone (C9/SKOV-3) were treated with CWHM-12 (10 μM) for 24h **(A).** The mRNA levels of EMT markers were assessed using qRT-PCR analysis. Data were normalized related to ACTB expression levels as an internal control. **(B)** migration and **(C)** invasion assays were performed with transwells as described in materials and methods. Photos represent one of the three independent experiments—original magnification: ×100. Results were presented as mean ± SD. *: $P < 0.05$, **: $P < 0.01$ and ***: $P < 0.001$ compared to untreated control cells (Ctrl), n=3. MCAs: Multicellular aggregates

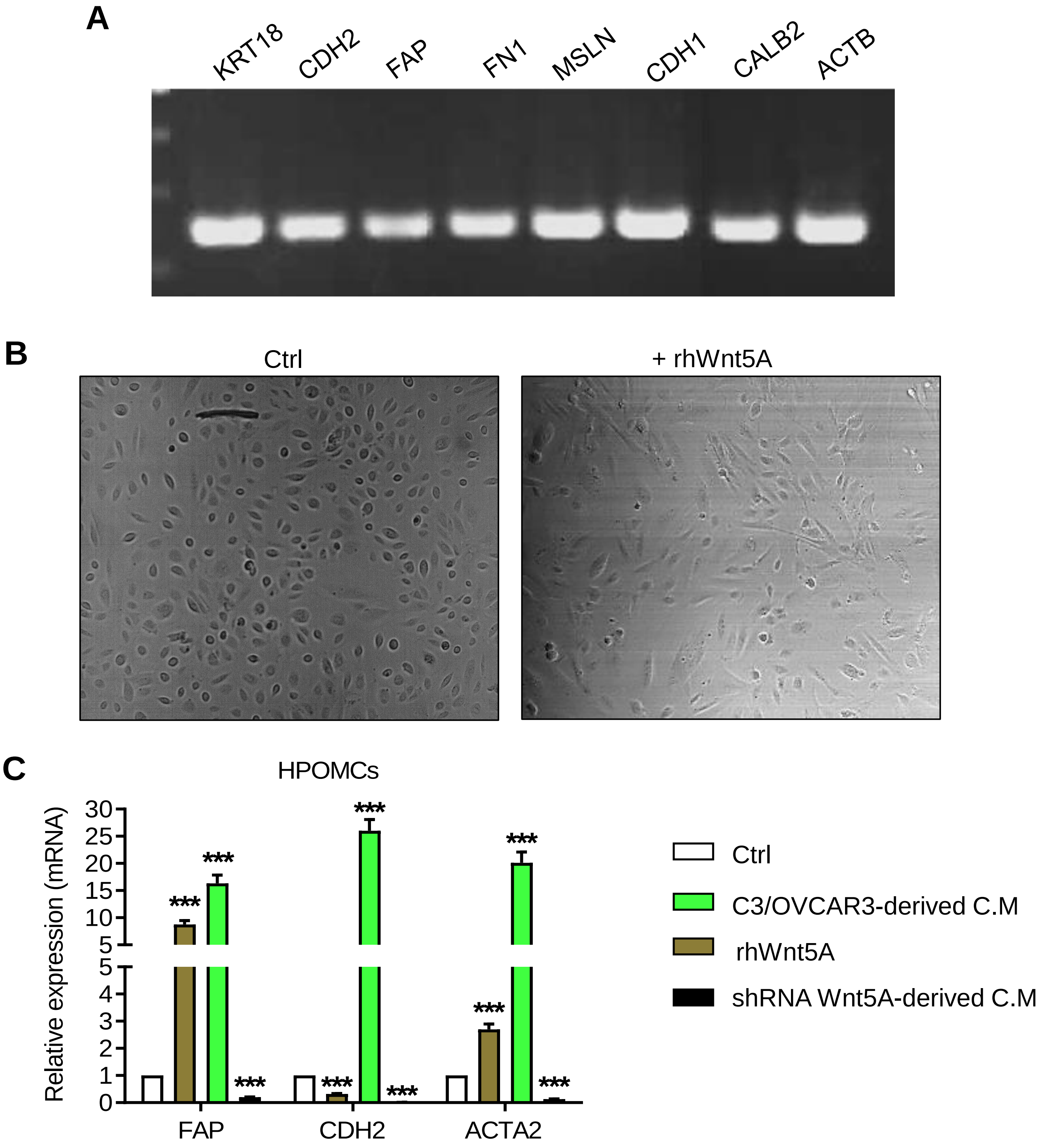

Figure S6. Characterization of human primary omental mesothelial cells (HPOMCs). **(A**) HPOMCs expresses mesothelial markers: Calbindin 2 (CALB2), Cytokeratin 18 (KRT 18), e-Cadherin (CDH1), Mesothelin (MSLN), N-cadherin (CDH2), fibronectin 1 (FN1), Vimentin (VIM) and lack Fibroblast activating protein (FAP) expression **(B)** phase contrast photos shows the phenotypic change of mesothelial cells from cobble-stone to spindle-like morphology in the presence of rhWnt5A (600 ng/ml). Original magnification: ×100. **(C)** The expression levels of EMT markers were assessed by qRT-PCR analysis. Data were normalized related to ACTB expression levels as an internal control and presented as mean ± SD. *: $P < 0.05$, **: $P < 0.01$ and ***: $P < 0.001$ compared to untreated control cells (Ctrl), n=3.
